# Remarks on Arsenic, Considered as a Poison and a Medicine; to Which Are Added, Five Cases of Recovery from the Poisonous Effects of Arsenic, Together with the Tests so Successfully Employed for the Detection of the White Metallic Oxide; in Which Those Satisfactory Methods Peculiar to Mr. Hume Were Principally Adopted, Confirmed, and Compared with Others Formerly in Use

**Published:** 1818-01

**Authors:** 


*Remarks on Arsenic, considered as a Poison and a Medicine; to which are added, five Cases of Recovery from the poisonous Effects of Arsenic, together with the Tests so successfully employed for the Detection of the white Metallic Oxide; in which those satisfactory Methods peculiar to Mr. Hume were principally adopted, confirmed, and compared with others formerly in Use.* By JOHN MARSHALL, Member of the Royal College of Surgeons, and Apothecary to His Royal Highness the Duke of Gloucester's Household. pp. 163. *London*, 1817.

IN a recent trial for murder by poison, we could not avoid blushing for our profession, in observing the contradictory and inconclusive evidence of the two medical gentlemen who attended the woman in her last moments. One, a veteran village practitioner; and the other, a young buck of the first fashion; who, we doubt not, would have made a most brilliant display of his knowledge of juridical medicine, had he been in the drawing room instead of a court of justice. It is, indeed, most astonishing, after the many examples which have occurred within these few years, that the study of juridical or forensic medicine has not become more general in this country, as nothing, surely, can be more overwhelming than the exposure of profound ignorance in the man, who, as in the instance now adduced, has been held up by his neighbours as possessing skill, talent, and ability. Yet we have, year after year, to lament the slow progress of this important branch of science, so necessary for the just and equable distribution of the laws by which we are protected. The administration of poison is so readily accomplished, and the crime is of such a demoniac nature, frequently extending far beyond the contemplation of the wretch himself; that it is most devoutly to be wished, that none should escape who may be guilty of a crime of such magnitude, at the same time, that the criminal should only be convicted on the clearest and most decisive evidence; and notwithstanding the surpassing "beauty, rapidity, and accuracy" of Mr. Hume's experiments for the detection of arsenic, we would caution our readers against a total reliance on them; and most strongly recommend its re-production in the metallic state, whenever it can be accomplished.

We hail every thing which may tend to the elucidation of this interesting subject with the highest satisfaction; and, as the communication of well authenticated cases is perhaps the very best mode of conveying medical intelligence, Mr. Marshall deserves our praise for his humane exertions, however we may differ from him in his general conclusions.

The volume now before us, is a republication of his former work on the Effects of Arsenic, and contains some additions which may be useful. In the prefatory introduction, we have a high eulogium on the experiments of Mr. Hume; and the general philanthropy of the author's disposition may be observed in the following sentiments:

"The pleasure of preserving human life, every professional gentleman derives in the course of practice; the agreeable and enviable sensation of satisfaction arising from such successful instances, is, if possible, more especially augmented, in all those cases wherein the functions of the vital powers, in the plenitude of health, are so suddenly assailed and liable to be extinguished, as by the rapid and destructive operation of poison."

The five cases described in Art. 1, are already well known; but as the knowledge of the symptoms arising from arsenic cannot be too widely diffused, our readers will excuse the following copious extracts.

*March* 21, 1815. "On entering the house I first saw Eliza Fenning,* the cook, lying on the stairs apparently in great agony,

* The unfortunate girl who afterwards suffered for the attempt to murder this family.

and complaining of a burning pain in the stomach, with violent retching, head-ache, and thirst. I directed her to drink some milk and water, and to be immediately conveyed to her bed; at the same time observing, that I would see her again as soon as possible, after having visited the rest of the family. My attention was then directed to Mr. Robert Turner, who appeared to be nearly in articulo mortis; his face, which had been swollen, having assumed the appearance of the true facies hippocratica, my apprehensions were considerable for his preservation. On examining the contents of the utensils in which he had vomited, a fluid was perceived of a yellowish and greenish colour, and in two of them stercoraceous matter. The pulse was gone; his voice faint and tremulous; and he pointed to the abdomen in great agony. On examination, I discovered a very remarkable irregularity of surface, occasioned by the spasmodic contractions of the muscles of the abdomen, and even of the viscera; this uneavenness extended from the epigastric region to the pubis, and to the right and left hypochondrium; and the excruciating pain was relieved for a short time by rubbing the abdomen with a piece of hot flannel and laudanum. From this state of the abdominal surface, there could be no doubt of the arsenic having gone far beyond the limits of the stomach into the alimentary canal. He complained of extreme faintness, and dreadful sickness. He had been violently purged; and on examination of the alvine secretions, the singularity of their appearance excited great surprise; they were all of a bright homogeneous green colour, like paint, and strongly resembled the green colour produced from a solution of the arsenic by one of Mr. Hume's tests, the amoniaco-sulphate of copper, which will afterwards be more fully described. Each effort of vomiting and purging was preceded and followed by these painful gripings and spasmodic contractions of the abdominal muscles. He complained of great heat in the stomach, which the patient compared to a furnace, or red hot irons; which sensation commenced at the tongue, and was felt throughout the course of the œsophagus to the cardia, or upper orifice of the stomach; insatiable thirst; violent head-ache; the eyes impatient of light; but the pupils sensible, and the extremities cold. The patient attempted in this dreadful state to get out of bed to walk to the night table; he was directly seized with vertigo, dimness of sight, and palpitation of the heart: he fell down, and went off into an epileptic fit; he was assisted on the bed, and in a few minutes recovered from the fit.

"Mrs. Robert Turner had great pain, and burning heat of the stomach; head-ache; immoderate thirst; vomiting and purging with olive green alvine discharges; tension of the abdomen; the face swollen; cold chills alternating with flushings of heat; and light was painful to the eyes. Her peculiar situation made me apprehensive of a miscarriage, in consequence of frequent bearing pains, more or less constant, in the loins; and, independently of these distressing symptoms, her mind was additionally agitated by the alarming state of her husband, who was lying by her side. If she had miscarried under these dreadful circumstances, there can be no hesitation in saying, she must have inevitably lost her life." p. 11—14.

Mr. Turner, senior, had symptoms similar to those already stated, but somewhat more moderate. The patients had been visited by Mr. Ogilvy, of Southampton Buildings, Chancery Lane, who had most judiciously cleansed the stomach, and administered some castor oil, with plenty of sugar and water, occasionally mixed with milk.

Mr. Gadsden, (Mr. Turner's apprentice) complained of a burning heat of the stomach; much nausea with vomiting; and severe gripings with purging: extreme faintness; palpitation of the heart; head-ache; and trembling of the right arm, and right lower extremities." p. 15—16.

It was resolved, that the purgative plan should be persisted in, and a full dose of castor oil was given, followed by a solution of the sulphas magnesiæ and manna, in mint water; and a saline effervescent draught was to be alternated every two hours with the salts, letting the alkali predominate, with the intention of neutralizing any remains of the arsenic, and relieving the disposition to vomit; and this system was to be continued until the alvine secretions became of a natural colour. The patients were allowed to drink frequently, and in small quantities, milk; soda water, with or without milk; and mutton broth. But these worthy citizens, who, perhaps, had never before been deprived of their favourite beverage, porter, felt extreme reluctance to comply with this simple regimen; and, in direct violation of the directions of Messrs. Marshall and Ogilvy, Mr. R. Turner could no longer restrain himself than the 25th, when he indulged freely in the use of porter and animal food, and, as might have been expected, he suffered severely for his imprudence, by the symptoms being greatly aggravated.

On the succeeding morning, Mrs. R. Turner's pulse was 130, and accompanied by faintness; but the bearing pain with the pain in the loins, had somewhat abated. They had all passed a most restless night.

"Mr. Gadsden appeared to be the most affected; he had been seized with four epileptic fits in the course of the night, preceded by a violent palpitation of the heart, accompanied with a peculiar tremulous action of the right arm and lower extremity; a considerable degree of symptomatic fever; insatiable thirst; a white but moist tongue; the face flushed; the respiration hurried; pulse 126, irregular and contracted; frequent gripings in the bowels, and spasmodic twitchings in the muscles of the chest and abdomen." p. 19.

"Mr. Robert Turner, in the early part of the morning, had another attack of epilepsy; the symptomatic fever ran high; the pulse 120; he complained of spasmodic twitchings about the chest and abdomen; palpitation of the heart; great languor, accompanied with a constant sensation of fainting; tongue white, but not dry; occasional chills, followed by an increase of heat; head-ache, and vertigo. A dose of the purgative mixture was administered, and the same medicine as on the preceding day continued." p. 19—20.

"The faces of all the four patients were swollen, with a fixed redness, more or less, under the eyes, and on the cheek bones; they had vomited two or three times in the course of the night by drinking too copiously of the diluents recommended over night, and each complained of the tongue and lips being sore and swollen.

"In the evening, the febrile symptoms had a little abated; the pain in the stomach was intense, occasionally remitting; and again returning with increased violence, with nausea and vomiting; much pain in the head; considerable thirst; and the bowels were open in each of the patients." p. 20.

"The saline draught with manna was to be continued, and the addition of camphor mixture."

On the 23d Mr. Gadsden received much relief from the camphire mixture, and asked for it often with eagerness; but he had violent head-ache, and was much agitated.

"On the 24th, they complained of a variety of singular nervous affections; tingling and burning sensations in the hands and feet in Mr. R. Turner, beginning at the extremities of the fingers, and gradually creeping to the shoulders; sometimes one foot, and at others, both affected with a burning feel, commencing at the toes, and gradually rising above the ankle joint; palpitation of the heart; great depression of the spirits, with a perpetual sensation of swooning; and frequent twitchings of the muscles of the chest and abdomen, and of the upper and lower extremities." p. 23.

"In the evening, they were all four evidently in a state of progressive convalescence; the febrile symptoms diminished; the pulse slower, less tremulous, and contracted; more natural and open in the beat. The pulse in Mrs. R. Turner continued at or about 100 for a fortnight afterwards." p. 24.

The state of Mrs. Turner naturally excited great anxiety and apprehension; but she was afterwards safely delivered of a very fine girl, who exhibited no appearance of injury from the violence of the poison. Mr. Gadsden was for a considerable time affected by epilepsy, and Mr. R. Turner had a singular sensation of numbness and prickling in the arms, accompanied by great weight for six weeks; this sensation was always removed by raising the arms.

The subject of Eliza Fenning has been so often discussed, that it will be unnecessary for us to enter upon it. Yet we can see nothing so very extraordinary in her reluctance to take medicine, or in her expressions that "she would as soon die as live." Nor can we admit this as a confirmation of her guilt. She has expiated her crime by an ignominious death, therefore let her soul rest in peace.

Art. 2, contains reflections on the natural and artificial causes which contributed to the recovery of the patients.

"These cases serve to illustrate the possibility of recovery after a considerable portion of arsenic has been taken; and in Mrs. R. Turner's case, under the most unfavourable of all circumstances during utero-gestation, and at the more critical period of near seven months: and their recovery was attributed to the speedy and spontaneous operation of the arsenic, both as a powerful emetic and purgative; and no doubt, to this joint operation the patients owed their immediate chance of escape from the imminent danger that awaited them; and by plentiful dilution, the stomach was wholly relieved and cleared from the deleterious particles; and the portion which escaped into the alimentary canal must have been carried from its surface by the powerful carthartic action of the poison. The severe injury which the arsenic had effected upon the coats of the stomach, the nervous system, and the circulating fluids, could not immediately cease, although the exciting cause had been effectually and promptly removed."

Mr. Marshall, in attributing the recovery to the spontaneous operation of the arsenic, agrees with Dr. Roget in his interesting observations on poison by arsenic, contained in the Medico-Chirurgical Transactions, vol. ii. He also conceives, with reason, that the peculiarly tough and horny composition in which the arsenic was incorporated, tended greatly to blunt the acrimony of the poison. This hardness cannot be attributed to the mixture of arsenic, as it is well known this mineral does not check the fermentative process.

Mr. Marshall considered the propriety of bleeding Mrs. R. Turner, and was prevented by the erroneous and unnecessary fear of producing too much debility. The disease induced by arsenic being administered, is admitted by all the best authors on the subject, such as Brodie, Orfila, Jaeger, Roget, Bostock, Renault, and Hill, to be of a highly sthenic nature; and can it be doubted that bleeding, under such circumstances, from a large orifice, would have accelerated her recovery, and greatly shortened the period of her convalescence? We repeat our opinion of such fears being groundless; and we cannot assent to the conclusion which he draws, that it might have done a great deal of harm. We agree most cordially in the propriety of using purgatives and diluents; and we have seen castor oil remain on the stomach, when every thing else has been rejected. The green colour of the alvine excretions is not so uncommon as Mr. Marshall imagines; we have frequently observed them in cholera morbus in warm climates; and though this colour may be attended to, we cannot admit of its being considered as a characteristic symptom of the presence of arsenic.

The singular alteration of appearance in Mr. R. Turner is worthy of remark. The countenance was of a completely yellow cast, and his features were so much altered and contracted, that his father scarcely knew him. When he re-entered the room, from whence he had withdrawn for the purpose of vomiting, he appeared strongly to resemble an aged man; so much so, that it was like a sudden metamorphosis from youth to age.

It is also worthy of notice, that they lost the entire skin of their tongues after they had taken the poison, and Mrs. Turner had the whole surface of the cuticle removed by degrees, in furfuraceous scales. We have frequently seen strangury induced by membraneous inflammation, therefore we cannot view this symptom as denoting the absorption of the arsenic into the circulating fluids, nor as affording any proof of its presence.

The remarks on epilepsy contain nothing new or interesting; but we may be permitted to observe, that had Mr. Gadsden been freely bled in the first instance, it is probable that the epileptic attacks to which he is still subject, might have been prevented or greatly modified.

Art. 3, contains some interesting cases of the effects of arsenic administered as a medicine. But we apprehend that most of the ill consequences arising from its use have been occasioned by the want of attention to previous evacuation, so judiciously recommended by Dr. Kellie and Mr. Hill, and by inattention to its earliest effects. We have seen constitutions of so peculiar an idiosyncrasy, that any mineral tonic would have produced similar effects. The cautions recommended, and the mode which Mr. Marshall proposes for the administration of this active mineral, so as to restrain its deleterious effects, are highly judicious; and we will venture to predict, that if they are attended to, the patient will neither become dropsical, nor paralytic, nor epileptic, nor blind!

Art 4, is taken up by some further observations, which merit attention; and states some successful cases of the administration of arsenic by Dr. Macqueen, of Norwich: but we must again urge the strong necessity of evacuation previous to its use; and we concur with Mr. Marshall, that while its application is restrained within bounds, no adverse symptoms will arise, nor will the patient be endangered by the severe attacks it is capable of exhibiting.

Art. 5, contains a strong recommendation of the carbonate of magnesia, as a remedy to counteract the deleterious effects of arsenic; but we would place much more reliance on the methods pursued by evacuants and diluents.

Art. 6, contains a variety of analytical experiments upon arsenic. That with the polished halfpenny is not so incontrovertible as Mr. Marshall would have us to believe. Phosphorus and zinc would emit the same alliaceous smell,* and mercury would leave a similar silvery whiteness. The tarnishing of the knives is too ridiculous to be advanced as a proof of the presence of arsenic; as, had the dumplings been ever so pure, the same effect would have been produced on them. The following mode of reproducing the white oxide is simple and easy:

"When white arsenic is mixed with carbonaceous or unctuous matter, and then exposed to a red heat in a glass tube closed at one end, it will be sublimed in the form of shining metallic scales; and then exposed to the air, under the heat of ignition, burn with a blue flame, and revert to the common white oxide." p. 84.

Yet we agree with our Author, that it is necessary to have a quantity that is quite tangible.

Our Author, in his zeal for the promulgation of Mr. Hume's tests, comes boldly forward to assert that they are preferred by the first chemists of the age to all others. We can readily suppose that Mr. Marshall and his apprentices prefer them; but we imagine the first chemists of the age would be as much pleased with those of Drs. Bostock, Roget, or Jaeger. Our limits will not permit us to enter deeply into their merits, but we shall make a few extracts, by which our Readers will judge for themselves.

"A small portion, hardly perceptible to the sight, of the powdered arsenic, was placed upon a piece of glass, and on this a single drop of the ammoniaco nitrate of silver was let fall; these were permitted to remain untouched for a few minutes, when the whole became yellow, of the same hue as the usual precipitate. This appeared more obvious when the glass was held over a piece

---

* See Jaeger's Theses, Edinburgh Medical and Surgical Journal, Vol. VII, p. 85.

of white paper, and still more so when it was removed and received upon the same paper.

"The ammoniaco nitrate of silver is fully capable of detecting even the 1000th part of a grain of the white oxide of arsenic in a state of solution; and cases, accompanied with suspicious circumstances, may possibly occur, wherein no portion of this substance could be otherwise detected." p. 94.

We had almost passed over a very delicate experiment which we shall now transcribe.

"A weak solution of the powder was made, by boiling it in a sand bath, in the proportion of about one grain to about four ounces of distilled water. About half an ounce of this solution was put into a phial, and a glass rod, previously dipped in the ammoniaco nitrate of silver, was applied to the surface. It instantly produced a fine yellow cloud, descending in an undulating form to the bottom of the phial, and gradually converted the transparent solution to an opaque or turbid yellow colour, and a copious precipitate was thus thrown down, which in the course of a few hours turned to a dark brown. This beautiful and highly satisfactory experiment infallibly proved the powder, which had been previously so well washed, to be white arsenic.

"The ammoniaco sulphate of copper was next applied, in the same manner, to another portion of the above solution of arsenic; and this was instantly changed into a flocculent and copious precipitate, which retained its bright green colour, forming the pigment so well known as Scheele's green." p. 89.

The methods which Mr. Hume recommends for preparing the ammoniaco nitrate of silver, and the ammoniaco sulphate of copper, may be interesting to many of our Readers.

"AMMONIACO NITRATE OF SILVER.

"Dissolve a few grains, say ten, of the nitrate of silver in about nine or ten times its weight of distilled water; to this add, by a drop at a time, some liquid ammonia, till a precipitate is formed; continue cautiously to add the ammonia, now and then shaking the bottle till the precipitate shall be nearly taken up, and the solution again become transparent, or nearly so, as the ammonia need not be in great excess, if in any; for solution of ammonia being lighter than water, the superfluous portion would be likely to remain on the surface of the fluid, to which this test-liquor is to be applied." p. 99.

"AMMONIACO SULPHATE OF COPPER.

"Let a little liquid ammonia be dropped into a saturated solution of sulphate of copper, until a precipitate be formed; then continue to add the ammonia by degrees; when, presently, the precipitate will be perfectly dissolved, and the solution will become of a rich, elegant, deep blue colour, and perfectly transparent." p. 101.

Art. 7, refers to the treatment, and is merely a repetition of what has been already advanced. Our author deprecates the advice to employ emetics in cases where acrid metallic poisons have been swallowed; and has favoured us with two interesting cases, in which the highest advantage was derived from sulphate of zinc, where lead had been accidentally swallowed, and when laudanum had been used for the purpose of self-destruction.

Art. 8, contains an abstract of the symptoms, and appears to us to be an unnecessary repetition.

Art. 9, is occupied with some experiments on human bile, for the purpose of proving that the green colour of the stools arose from a chemical change in the biliary secretion. In our experiments on bile, no change has been observed from the mixture of arsenic; and we have already stated, that we have seen the stools of the same colour when no arsenic has been used.

Art. 10, is devoted to a case of Cancer, which, it is alleged, was much benefited by the application of arsenic, in the form of an ointment. We can, however, see nothing to recommend it over many other escharotics; and when it is taken into consideration, that we have some well authenticated cases of this mineral being absorbed, and even causing death, we would caution our readers against its use.

Art. 11, contains remarks on the dangerous effects of arsenious oxide, used in the form of a lotion, which exhibit nothing new or very interesting; we certainly concur in the great danger which may arise from its use.

Art. 12, is a practical application of the chemical analysis; and contains some useful hints to guide us in our investigation, either antecedent or posterior to dissolution.

The Appendix is the result of the Author's experiments on the decoction of onions, which he commenced in consequence of the conflicting medical evidence delivered in a recent trial at Launceston in Cornwall. The results are clear and conclusive, and we recommend them to the perusal of all who may have a desire for further information on this interesting subject.

Before we take leave of our author, we cannot but express how much we lament that he has not paid more attention to the stile and composition of his book. We are not advocates for "splendour of diction, brilliancy of conception," or elegance of composition, superseding the simple narration of facts; but it is the duty of every man of a liberal education, particularly when he presents himself to the public, to write intelligibly and grammatically. Now the orthographical and grammatical errors, which appear in almost every chapter of the work before us, whether they arise from inattention or not, are disgraceful to a gentleman of a liberal profession. The Latinity of the prescriptions also, would cause a blush on the cheeks of a school-boy; and we can scarcely comprehend what is intended by "the high state of irritable excitement to which the *organ of the stomach* is reduced." We have no doubt our Author will receive these hints with the same good humour they are intended; and that in a subsequent edition we shall have the pleasure of observing the errors of the present volume expunged and rectified.

---

*An Elementary Summary of Physiology.* By F. Majendie, Doctor of Medicine of the Faculty of Paris; &c. &c.

